# Cognitive functions of the posterior parietal cortex: top-down and bottom-up attentional control

**DOI:** 10.3389/fnint.2012.00038

**Published:** 2012-07-04

**Authors:** Sarah Shomstein

**Affiliations:** Department of Psychology, George Washington University, WashingtonDC, USA

**Keywords:** attention, bottom-up attention, capture, inferior parietal lobule (IPL), parietal cortex, superior parietal lobule (SPL), temporo-parietal junction (TPJ), top-down attention

## Abstract

Although much less is known about human parietal cortex than that of homologous monkey cortex, recent studies, employing neuroimaging, and neuropsychological methods, have begun to elucidate increasingly fine-grained functional and structural distinctions. This review is focused on recent neuroimaging and neuropsychological studies elucidating the cognitive roles of dorsal and ventral regions of parietal cortex in top-down and bottom-up attentional orienting, and on the interaction between the two attentional allocation mechanisms. Evidence is reviewed arguing that regions along the dorsal areas of the parietal cortex, including the superior parietal lobule (SPL) are involved in top-down attentional orienting, while ventral regions including the temporo-parietal junction (TPJ) are involved in bottom-up attentional orienting.

## Introduction

Successful interaction with our sensory environment requires an intricate balance of two attentional selection mechanisms—that of top-down and bottom-up. Heading over to the produce aisle of your local supermarket with the goal of picking up few needed ingredients for the mango salad, engages deployment of voluntary goal-directed, or top-down, attentional system such that you actively search for all the required ingredients among the multitude of produce choices. However, should you hear a ringer of a cell phone, it will most likely capture your attention and interrupt your search. Such interruption occurs in a bottom-up, or stimulus-driven, fashion whereby a mere salience of the stimulus, the fact that the ring is different from other sounds in your environment, deems it worthy of selection. The described scenario underscores the importance of goal-directed and stimulus-driven selection for behavior, and points to a fine balance that has to exist between the two attentional systems to prevent “tunnel vision” on the one hand and complete inability to focus on the other.

## Top-down and bottom-up selection: behavior

Several decades of behavioral research have been dedicated to demonstrating that the distribution of attention can be controlled by intentions of the observer as well as by the salience of the physical stimulus. Much of behavioral evidence for top-down and bottom-up attentional allocation has been reviewed extensively elsewhere (Johnston and Dark, 1986; Egeth and Yantis, 1997). To summarize, studies demonstrating effects of top-down attentional control show that attention can be successfully allocated to spatial locations, features, objects, etc., following presence of exogenous or endogenous cues (Eriksen and Hoffman, 1972; Posner, 1980; Posner et al., 1980), or expectations either set by prior knowledge or by contingencies of the stimulus (Shaw, 1978; Moore and Egeth, 1998; Geng and Behrmann, 2002, 2005; Shomstein and Yantis, 2004a; Drummond and Shomstein, 2010). Evidence supporting bottom-up attentional allocation has relied on various attentional capture paradigms, in which participants are engaged in a top-down search and their attention is diverted to the task-irrelevant stimuli, demonstrating that attention is captured by feature singletons (unique item; Yantis and Jonides, 1990; Theeuwes, 1991; Folk et al., 2002) and abrupt onsets (Yantis and Jonides, 1984; Theeuwes, 1991; Koshino et al., 1992; Juola et al., 1995).

Whereas most early studies concentrated on demonstrating evidence for top-down and bottom-up attentional selection, most recent studies shifted their focus to examining how the two attentional selection systems interact. This line of investigation is fueled by observations that in order to effectively select task-relevant information (e.g., ingredients for the salad) one must actively inhibit the task-irrelevant information that would otherwise divert attention away from the task at hand. The flip side of this logic, is that the less one is focused on task-related information the more capture will ensue. It has been shown experimentally that the attentional state of the observer predicts what type of information, and to what extent, will ultimately capture attention (Folk et al., 1992, 2002; Bacon and Egeth, 1994; Gibson and Kelsey, 1998). For example, Folk et al. (2002) showed that when searching for a red letter, an observer will be more readily captured by an irrelevant stimulus in the periphery if that stimulus is red, or matches the target template in some way. Since the observer's top-down control settings are set to search for a red feature, any stimulus that is red is likely to capture attention and potentially interfere with top-down control. Thus, with a capture task, attentional search strategies can be distinguished from one another by varying the similarity levels between the stimulus properties of the target and distractors. The more similar the target is to the distractor, the more difficult it is for the observer to avoid capture.

## The role of the parietal lobe in top-down and bottom-up selection: neuroimaging

**Figure 1:**
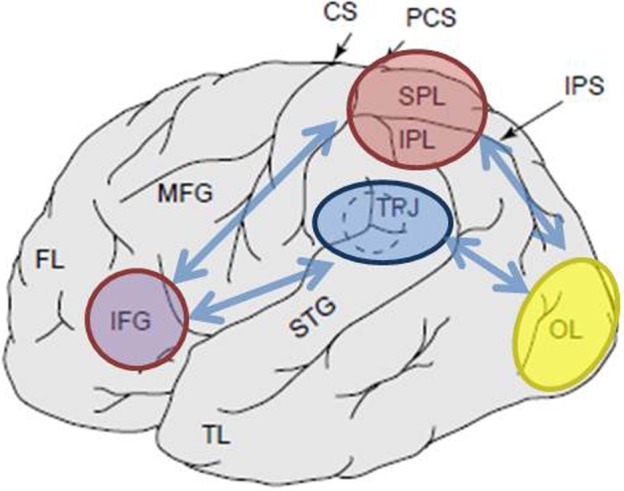
**Schematic depiction of relevant anatomical landmarks projected onto the lateral surface of the human brain.** Superior parietal lobule (SPL) and inferior parietal lobule (IPL) are regions within the dorsal part of the parietal cortex subserving top-down attentional orienting. Temporo-parietal junction (TPJ) is a region within the ventral parietal cortex subserving bottom-up attentional orienting. Both, SPL and TPJ, are thought to elicit control signals responsible for subsequent attentional modulations observed over sensory regions, in this case modulating (labeled with dark blue arrows) visually evoked activity in the occipital lobe (OL). Additionally, areas along the inferior frontal gyrus (IFG) and inferior frontal junction (IFJ) are thought to serve as convergence areas for stimulus-driven and top-down attentional control (marked by light blue bi-directional arrows).

Various neuroimaging techniques provided strong evidence for the involvement of parietal cortex in top-down and bottom-up orienting, with the evidence reviewed extensively elsewhere (Corbetta and Shulman, 2002, 2011; Behrmann et al., 2004). It has been demonstrated that areas most commonly activated following top-down cues to attend to particular locations, features, or objects are located along the dorsal parts of the parietal cortex. Such areas include inferior parietal lobule (IPL), dorsomedial regions referred to as superior parietal lobule (SPL), as well as more medial regions along the precuneus gyrus (Yantis et al., 2002; Giesbrecht et al., 2003; Liu et al., 2003; Yantis and Serences, 2003; Figure 1). Several top-down tasks have been shows to successfully engage dorsal regions of the parietal cortex, namely those involving spatial (Kastner et al., 1999; Corbetta et al., 2000; Hopfinger et al., 2000; Shomstein and Behrmann, 2006; Chiu and Yantis, 2009; Greenberg et al., 2010) as well as non-spatial shifts of attention (Giesbrecht et al., 2003; Yantis and Serences, 2003; Shomstein and Yantis, 2004b, 2006; Tamber-Rosenau et al., 2011).

In a typical task aimed to engage the top-down attentional allocation, individuals are shown two rapid serial visual presentation (RSVP) streams positioned peripherally and are initially instructed to monitor one stream for a cue (e.g., a digit among the stream of letters). The identity of the cue indicates whether the subject must maintain attention on the current stream or shift attention to the other stream (Yantis et al., 2002; Yantis and Serences, 2003). Two major findings are observed in such paradigms. The first has to do with increased activation within the sensory regions representing the at-the-moment attended location (e.g., increased activity within the left primary visual regions when the right RSVP stream is attended). This finding provides firm evidence that participants are attending to a specific location and that attention modulates the strength of the sensory response (see Figure 1; Moran and Desimone, 1985; O'Craven et al., 1997). The second finding has to do with the observation that dorsal regions of the parietal lobe are selectively activated by shifts of top-down attention. It is observed that the SPL/IPL timecourse of activity is transient in nature suggesting that this area of the parietal cortex is the source of a brief attentional control signal to shift attentive states in a top-down manner (Yantis et al., 2002).

Several fMRI studies have documented that bottom-up attentional capture, mediated by stimulus salience and/or relevance, is subserved by the temporo-parietal junction (TPJ; Figure 1). For example, when subjects attend to and monitor a change in either a visual or auditory stimulus, presented simultaneously, activation of the TPJ regions of the parietal lobe is enhanced. In addition to the apparent sensitivity to relevant stimuli, TPJ is also activated in response to potentially novel (unexpected or infrequent) events when an organism is engaged in a neutral behavioral context or when engaged in a task (Marois et al., 2000; Downar et al., 2002; Serences et al., 2005; Corbetta et al., 2008; Asplund et al., 2010; Diquattro and Geng, 2011; Geng and Mangun, 2011). This activation occurs independent of the modality (auditory, tactile, and visual) in which the input is delivered, reflecting multisensory nature of TPJ (but see Downar et al., 2001).

In a typical task examining the neural mechanism of bottom-up attentional capture, participants are presented with an RSVP stream of items in the center of the display and are asked to identify a pre-defined target (e.g., identify red letter presented within an RSVP stream of white non-targets). Some proportion of trials contains a task-irrelevant salient distractor presented at various time intervals prior to the onset of the target, while other trials contain only the salient distractor (i.e., without the target). “Target-distractor” trials are used in order to assay the extent of capture, showing that the task-irrelevant distractor is in fact salient thereby yielding a decrease in target accuracy. The “distractor-in-isolation” trials are used for further analyses since such trials allow for the examination of activity elicited to the salient distractor without contamination from the target-related processes. Several important findings emerge from such paradigms. First, when distractors are spatially separated from the target location, capture distractors are accompanied by increased cortical activity in corresponding regions of the sensory cortex (e.g., retinotopically organized visual cortex; see Figure 1). Such results provide strong evidence that during capture, spatial attention is in fact captured to the spatial location occupied by the distractor (Serences et al., 2005). Second, ventral regions of the parietal cortex, mainly within the TPJ are selectively activated by bottom-up, involuntary, shifts of attention. Just as activity within the SPL for the top-down orienting, the timecourse of activity observed over TPJ is transient in nature suggesting that this region is the source of a brief attentional control signal to shift attention in a bottom-up manner.

It should be noted that while this review is focused on addressing cognitive functions of the posterior parietal cortex, other regions, notably those within the frontal cortex are also recruited for top-down and bottom-up attentional allocation. Such regions include the ventral frontal cortex (VFC), the frontal eye fields (FEF), inferior frontal junction (IFJ), and inferior frontal gyrus (IFG; Corbetta and Shulman, 2002, 2011; Serences et al., 2005; Asplund et al., 2010; Diquattro and Geng, 2011).

## The role of the parietal lobe in top-down and bottom-up selection: neuropsychology

Historically researchers relied critically on neuropsychological studies of patients with hemispatial neglect (a disorder of spatial allocation of attention to the left hemi-space) to gain insight into cognitive functions associated with the parietal lobe. In the classical neuropsychological literature, parietal cortex, as an entirety, was generally considered the primary lesion site for hemispatial neglect. This view, elaborated in detail by early researchers (Critchley, 1953; McFire and Zangwill, 1960; Piercy, 1964) clearly recognized the association between the parietal lesion and the ensuing neglect. This perspective was largely held through the 1980s when Posner and colleagues (1984) used the covert visuospatial cueing paradigm to show that damage to the parietal lobe produces a deficit in the “disengage” operation (retracting attention from one location and shifting it to another) when the target is contralateral to the lesion. However, despite this major advance in understanding the neural basis of attention and specifically the “disengage” role of parietal cortex, their findings assume a single cortical site (parietal cortex) and a single functional capability (“disengage”). In contrast with this more monolithic approach to the brain (parietal cortex) and behavior (attentional disengagement), recent behavioral and neuroimaging work (reviewed above and elsewhere) suggests that both the cortical region and the associated attentional behavior may be subdivided into qualitatively different profiles.

Given segregation of the cortical networks into top-down and bottom-up processes, an obvious prediction is that damage to superior portions of the parietal lobule (subsuming SPL) should yield a deficit in goal-directed attentional orienting, whereas damage to the inferior portions of the parietal lobule (subsuming TPJ) would result in a deficit associated with stimulus-driven attention capture. To the extent that these brain-behavior correspondences have been explored in the neuropsychological literature, this prediction is not obviously upheld. For example, clinical symptoms of hemispatial neglect are strongly associated with damage to the inferior portions of the parietal lobe, which includes TPJ, rather than to superior portions like SPL (Friedrich et al., 1998; Shomstein et al., 2010; Corbetta and Shulman, 2011). This is somewhat at odds with the neuroimaging literature, which suggests that the role of TPJ is in the capture of attention, rather than in the voluntary orienting of attention, the domain in which neglect patients seem to have the most difficulty. To complicate matters further, it has been noted that lesions that involve SPL exclusively, only rarely produce clinical evidence of neglect (Vallar and Perani, 1986). Another recent study with patients with lesions centered primarily over TPJ and STG but preserved SPL, Corbetta et al. (2005) showed that spatial neglect, as well as its recovery, was associated with restoration of activity in *both* the ventral temporo-parietal and dorsal parietal regions (see Corbetta and Shulman, 2011 for a review). While interesting and exciting in its conclusions, this last study does not differentiate the relative contribution of dorsal and ventral pathways to different types of attention, since patients were only tested on a variant of the Posner covert spatial attention cuing task, task that is thought to engage both top-down and bottom-up attentional orienting.

To distinguish between goal-driven attentional control and salient attentional capture and to examine their mapping onto the SPL and TPJ, respectively, recent study adopted two behavioral paradigms, each targeting one of these forms of attention (Shomstein et al., 2010). To examine the integrity of top-down attentional orienting in the patients, a top-down task was used requiring participants to shift spatial attention between the spatially separated RSVP streams (a task that has been successfully used to demonstrate SPL activation in fMRI studies (Yantis et al., 2002)). Similarly, in order to examine the bottom-up attentional orienting abilities of the patients, a variant of Folk et al. (2002) contingent capture paradigm was employed in which participants detected targets that appeared at fixation while task-irrelevant color singletons were flashed in the periphery. The extent to which task-irrelevant distractors interfere with the central detection task was then used as a measure of bottom-up attentional capture (Bacon and Egeth, 1994; Folk et al., 2002).

**Figure 2:**
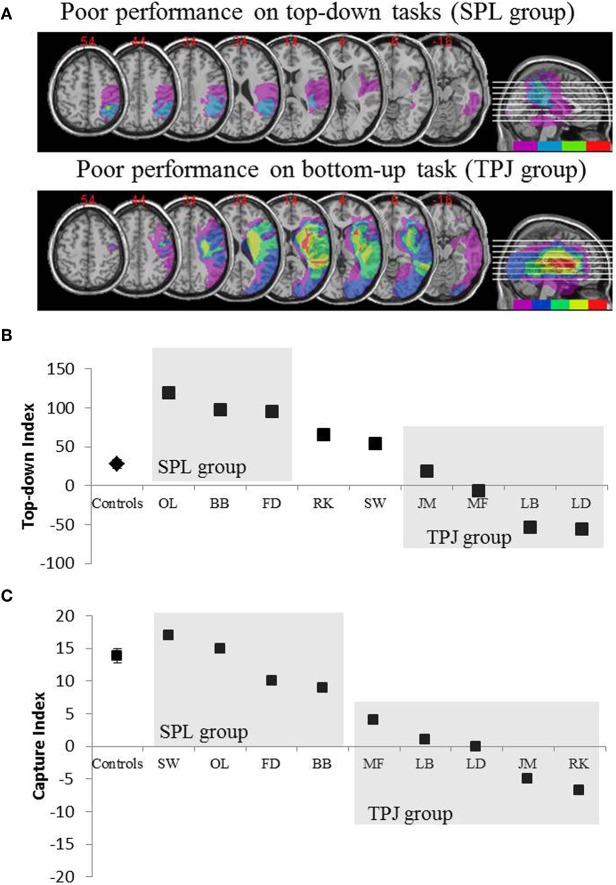
**Results of the neuropsychological study aimed at investigating the relative contribution of SPL and TPJ to top-down and bottom-up orienting. (A)** Lesion overlaps (purple minimal overlap; red maximal overlap) for patients grouped by behavioral deficits in top-down attentional orienting, labeled the SPL group (top panel); and patients grouped by behavioral deficits in bottom-up orienting, labeled the TPJ group (lower panel). **(B)** Behavioral performance on the top-down task summarized with a “Top-down Index” which quantifies differences between spatial top-down shifts made from left to right and vice versa. Controls and the TPJ lesioned group show similar efficiencies in executing spatial shifts, while patients with SPL lesions show decreased efficiency. Group control and individual patient data (labeled with patient initials) are plotted on the abscissa. **(C)** “Capture index” is a measure of bottom-up attention and quantifies the extent to which task-irrelevant distractors capture attention away from the task. Controls and the SPL lesioned group show similar capture values, such that both groups are captured by the task-irrelevant distractors. TPJ lesioned group show much reduced capture index (failure to be captured). Note that patients were placed in the SPL or TPJ group based on behavior, rather than based on the lesion, thus note the consistency with which patients end up in the corresponding group.

The predictions were as follows: patients with lesions to superior portions of the parietal lobe (affecting SPL) should be impaired in the top-down attentional orienting task (with preserved performance on the capture task) while patients with lesions to the inferior portions of the parietal lobe (affecting TPJ) should be impaired on the capture task (with spared performance on the top-down task). A double dissociation of this form not only attests to the independent components of attention but also suggests that such attentional components are mediated by independent neural mechanisms. Eight patients with visuo-spatial neglect were recruited for the study and completed two tasks, tapping either stimulus-driven or goal-directed attentional orienting. Based on their behavioral profile, patients were sorted into groups and their lesion overlap was explored (Figure 2A). Patients who exhibited difficulties with goal-directed attentional orienting, as quantified by the top-down attentional index (Figure 2B), presented with lesion overlap centered over superior portions of the parietal lobule (subsuming SPL) with spared inferior parietal lobule (TPJ). Patients with lesion overlap centered over the inferior portions of the parietal lobule (subsuming TPJ) but spared SPL performed normally on the goal-directed orienting task, while remaining immune to attentional capture (Figure 2C). The findings from this study clearly suggest that SPL and TPJ are anatomical regions that are necessarily recruited for the purposes of top-down and bottom-up orienting and that damage to SPL and TPJ leads to disorders of top-down and bottom-up orienting respectively.

## Interaction between top-down and bottom-up selection

Although there is apparently a strong association between goal-directed orienting and SPL and stimulus-driven orienting and TPJ, data from Shomstein et al. (2010) patient study suggest that these two systems are not entirely independent. This conclusion is supported by the finding that patients with SPL damage exhibited a pattern of performance labeled as “hyper capture.” Unlike controls, for whom only target colored distractor captured attention (leading to lower target accuracy), irrelevant colored distractors also proved to be distracting for patients with SPL lesion. In addition, whereas for controls attention was captured by distractors only when they preceded the onset of the target, for patients with SPL lesions attention was even captured by distractors presented simultaneously with the target. This pattern of performance can be explained by the following framework: SPL is responsible for top-down guidance of attention that includes determining the aspects of the stimuli that are task relevant (e.g., search for red target; Corbetta and Shulman, 2002; Serences et al., 2005). This attentional set then constrains TPJ, such that the capture of attention mechanism that is mediated by TPJ is only triggered by the task relevant information (e.g., red distractors capturing attention, and gray distractors not capturing attention when searching for a red target). The absence of SPL prevents the establishment of a task relevant attentional set and thus any stimulus, task relevant or not, is deemed important therefore capturing attention (e.g., task-irrelevant distractor capturing attention for the SPL group) indiscriminately.

It has been suggested that SPL and TPJ could interact in at least one of two possible ways. The first possibility is that TPJ serves as an alerting system that detects behaviorally relevant stimuli but lacks the high spatial resolution, thus when a behaviorally relevant stimulus is detected its precise location is supplied by the SPL that stores spatial maps (Kastner et al., 1999; Wojciulik and Kanwisher, 1999; Bisley and Goldberg, 2003; Silver et al., 2005). A related hypothetical possibility is that the capture mechanism (that includes TPJ) acts as a circuit breaker of ongoing cognitive activity when a behaviorally relevant stimulus is presented (Corbetta and Shulman, 2002, 2011). The “hyper-capture” pattern of activity observed in patients with preserved TPJ but lesioned SPL provides further evidence for the hypothesis that views TPJ as issuing a control signal that terminates the task at hand thus serving as a circuit breaker (Corbetta and Shulman, 2002; Serences et al., 2005). Other recent neuroimaging studies employing various paradigms have provided further evidence for an interactive relationship between the top-down and the bottom-up attentional orienting, and subsequently for the relationship between SPL and TPJ (Serences et al., 2005; Asplund et al., 2010; Diquattro and Geng, 2011).

While the evidence for an interaction between the two attentional systems and the two attentional substrates (SPL and TPJ) is strong, what remains unclear is whether this interaction is direct between SPL and TPJ or whether it is accomplished through other intermediary regions. As was mentioned earlier, top-down and bottom-up attentional orienting networks engage various regions within the frontal cortex, thus it is reasonable to hypothesize that the convergence between the two systems might be accomplished via the frontal lobe. Two recent studies investigating the interaction between top-down and bottom-up attentional selection provided evidence for the IFJ and IFG as possible sites of convergence between stimulus-driven and goal-directed selection (Asplund et al., 2010; Diquattro and Geng, 2011). The IFJ and IFG appear to be ideal candidates for such interaction given their general involvement in attention and cognitive control as well as its involvement in both spatial and non-spatial selection (Koechlin et al., 2003; Brass et al., 2005).

## The role of the parietal lobe in top-down and bottom-up selection: physiology

While the emphasis of this review has been predominantly placed on human studies, a great wealth of knowledge about the involvement of parietal cortex in attentional orienting has been gleaned from monkey physiology investigations (see recent review by Bisley and Goldberg, 2010). However, when it comes to examining the relative contributions of different regions within the parietal cortex to top-down and bottom-up attentional orienting, monkey physiology literature falls short. The primary reason for this is that within the monkey cortex there does not appear to be evidence for the same segregation of top-down and bottom-up control. Instead, lateral intraparietal area (LIP) originally thought to be involved in saccade planning (Gnadt and Andersen, 1988) is involved in visual attention and acts as a priority map in which external stimuli are represented according to their behavioral priority derived in either top-down or bottom-up manner (Colby and Goldberg, 1999; Bisley and Goldberg, 2003, 2010; Balan and Gottlieb, 2006; Ipata et al., 2006; Buschman and Miller, 2007; Gottlieb and Balan, 2010).

## Conclusion

Although much less is known about human parietal cortex than that of homologous monkey cortex, recent studies, employing neuroimaging and neuropsychological methods, have begun to elucidate increasingly fine-grained functional and structural distinctions. This review focused on recent neuroimaging and neuropsychological studies elucidating the cognitive roles of dorsal and ventral regions of parietal cortex in top-down and bottom-up attentional orienting, and on the interaction between the two attentional allocation mechanisms.

### Conflict of interest statement

The author declares that the research was conducted in the absence of any commercial or financial relationships that could be construed as a potential conflict of interest.
